# Decreased risk of dementia in migraine patients with traditional Chinese medicine use: a population-based cohort study

**DOI:** 10.18632/oncotarget.19094

**Published:** 2017-07-08

**Authors:** Chun-Ting Liu, Bei-Yu Wu, Yu-Chiang Hung, Lin-Yi Wang, Yan-Yuh Lee, Tsu-Kung Lin, Pao-Yen Lin, Wu-Fu Chen, Jen-Huai Chiang, Sheng-Feng Hsu, Wen-Long Hu

**Affiliations:** ^1^ Department of Chinese Medicine, Kaohsiung Chang Gung Memorial Hospital and School of Traditional Chinese Medicine, Chang Gung University College of Medicine, Kaohsiung, Taiwan; ^2^ School of Chinese Medicine for Post Baccalaureate, I-Shou University, Kaohsiung, Taiwan; ^3^ Department of Physical Medicine and Rehabilitation, Kaohsiung Chang Gung Memorial Hospital and Chang Gung University College of Medicine, Kaohsiung, Taiwan; ^4^ Department of Neurology, Kaohsiung Chang Gung Memorial Hospital and Chang Gung University College of Medicine, Kaohsiung, Taiwan; ^5^ Department of Psychiatry, Kaohsiung Chang Gung Memorial Hospital and Chang Gung University College of Medicine, Kaohsiung, Taiwan; ^6^ Department of Neurosurgery, Kaohsiung Chang Gung Memorial Hospital, Kaohsiung, Taiwan; ^7^ Management Office for Health Data, China Medical University Hospital, Taichung, Taiwan; ^8^ College of Medicine, China Medical University, Taichung, Taiwan; ^9^ Graduate Institute of Acupuncture Science, China Medical University, Taichung, Taiwan; ^10^ Department of Chinese Medicine, China Medical University Hospital, Taipei Branch, Taipei, Taiwan; ^11^ Kaohsiung Medical University College of Medicine, Kaohsiung, Taiwan; ^12^ Fooyin University College of Nursing, Kaohsiung, Taiwan

**Keywords:** dementia, migraine, pharmaco-epidemiology, national health insurance research database, Chinese herbal product

## Abstract

Patients with migraine are reportedly at increased risk of developing dementia. We aimed to investigate the association between traditional Chinese medicine (TCM) use and dementia risk in migraine patients. This longitudinal cohort study used the Taiwanese National Health Insurance Research Database to identify 32,386 diagnosed migraine patients aged 20 years and above who received treatment from 1997 to 2010. To balance comparability between TCM users and non-TCM users, we randomly selected equal numbers from each group, and compared subgroups compiled based on combinations of age, sex, index year, and year of migraine diagnosis. All enrollees received follow-up until the end of 2013 to measure dementia incidence. We identified 1,402 TCM users and non-TCM users after frequency matching. A total of 134 subjects were newly diagnosed with dementia during the follow-up period. TCM users were significantly less likely to develop dementia than non-TCM users. The most frequently prescribed formulae and single Chinese herbal products were Jia-Wei-Xiao-Yao-San and Yan-Hu-Suo, respectively. This population-based study revealed a decreased dementia risk in migraine patients with TCM use. These findings may provide a reference for dementia prevention strategies, and help integrate TCM into clinical intervention programs that provide a favorable prognosis for migraine patients.

## INTRODUCTION

Migraine is a primary headache disorder characterized by recurrent episodes of moderate to severe pulsating headache, most often unilateral, that is aggravated by physical activity and associated with nausea, photophobia, or phonophobia [1]. Reports of migraine prevalence vary broadly. The reported prevalence of migraine is 15% in Europe, 13% in North America, 9% in Asia, and 5% in Africa [2]. Migraine can affect all age groups, but it most commonly occurs in females and those aged from 25 to 55 years old, the peak years of economic productivity [3]. Recently updated statistics from US government health surveillance studies show that migraine remains a highly prevalent medical condition, affecting approximately 15% of Americans annually [4].

Dementia is characterized by slow progressive deterioration in memory and cognitive function, and an inability to perform personal daily activities [5]. It is a neurodegenerative disorder that typically affects older people. With the global aging population, the number of patients with dementia will rise and place an increasing burden on families and the healthcare system [5]. A recent epidemiologic study has demonstrated that migraine is associated with an increased risk of developing dementia [6]. Thus, how to prevent the occurrence of dementia in patients with migraine is an essential public health issue.

Traditional Chinese medicine (TCM) is a form of complementary and alternative medicine (CAM) that has been widely applied for centuries in Asian countries. Since 1995, Chinese herbal products (CHPs) have been listed under the National Health Insurance (NHI) program, which is a government-run, single-payer program that covers more than 99% of Taiwanese citizens and over 93% of Taiwanese healthcare institutes [7]. In Taiwan, like Western medicine, TCM is widely used for the treatment of migraine. Increasing evidence suggests that reducing modifiable risk factors such as smoking, midlife hypertension, midlife obesity, and diabetes may reduce the prevalence of dementia [8]. In addition, some potentially protective medications for dementia have been reported such as antihypertensive drugs, statins, hormone replacement therapy, and non-steroidal anti-inflammatory drugs [9]. However, to the best of our knowledge, no studies have investigated the use of TCM for reducing the risk of dementia in migraine patients.

This population-based cohort study aimed to investigate the risk of dementia development in migraine patients with or without TCM use. We also identified the most commonly used CHPs in migraine sufferers.

## RESULTS

**Figure 1:**
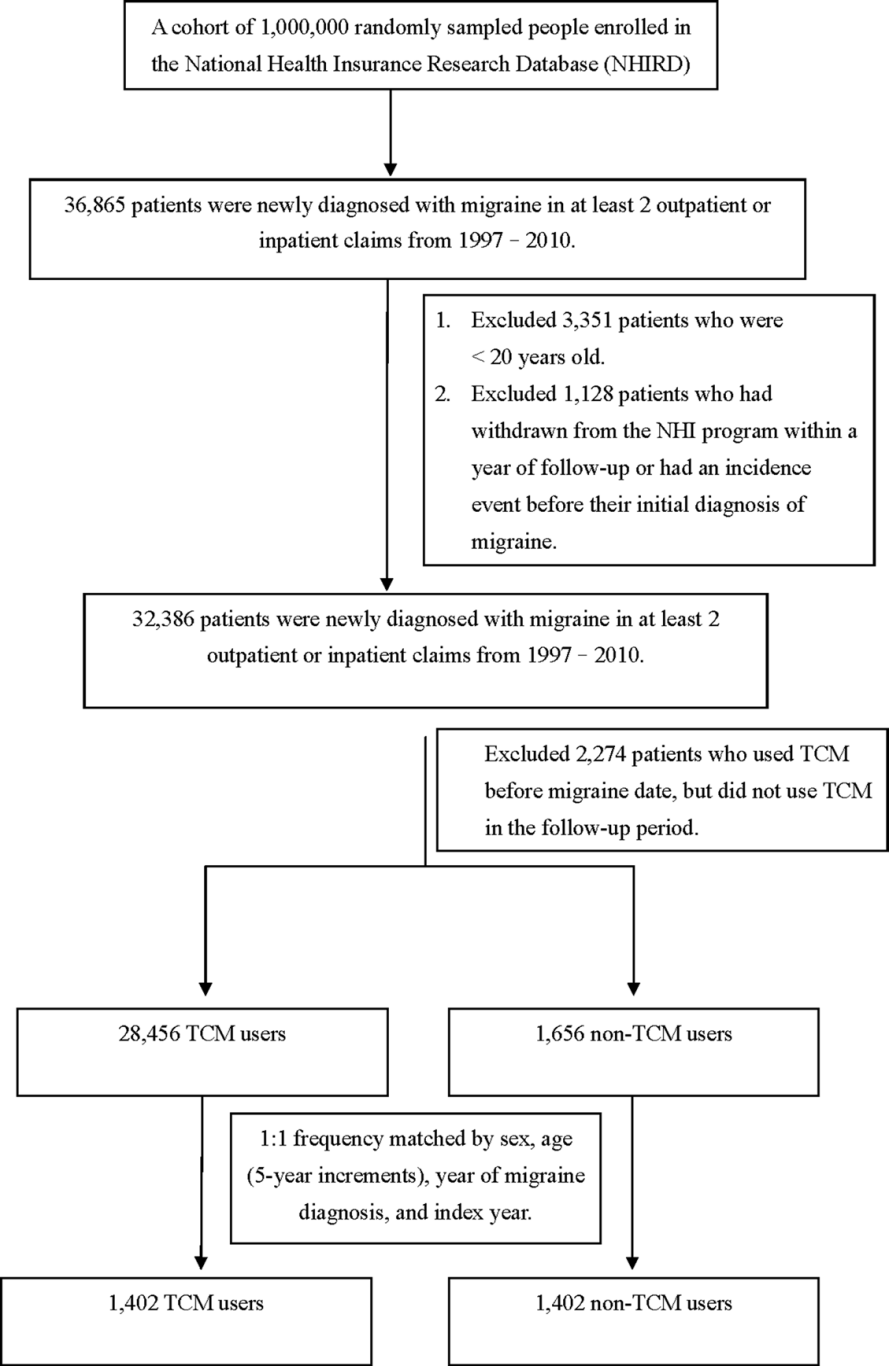
The participant selection process in the study and comparison cohorts

Using data from January 1997 to December 2010, we identified 1,402 TCM users and 1,402 non-TCM users after frequency matching (Figure 1). Table 1 shows baseline characteristics of the migraine patients in the TCM and non-TCM groups. The mean ages were 51.20 (standard deviation [SD], ± 16.57) years and 51.32 (SD, ± 16.61) years for the TCM and non-TCM users, respectively. The percentages of females and males were 50.43% and 49.57%, respectively. Compared with non-TCM users, TCM users had significantly higher proportions of comorbidity with hyperlipidemia. The mean follow-up periods were 7.00 (median = 6.31) and 5.56 (median = 4.91) years for the TCM and non-TCM groups, respectively.

**Table 1:** Characteristics of migraine patients according to use of TCM and non-used

Variable	Migraine patients				*P*-value
	TCM				
	No (*n =* 1402)		Yes (*n =* 1402)		
	*n*	%	*n*	%	
**Sex**					0.99^*^
Female	695	49.57	695	49.57	
Male	707	50.43	707	50.43	
**Age group**					0.99^*^
20–29	165	11.77	165	11.77	
30–39	229	16.33	229	16.33	
40–49	307	21.9	307	21.9	
50–59	259	18.47	259	18.47	
60–69	191	13.62	191	13.62	
70–79	200	14.27	200	14.27	
More than 80	51	3.64	51	3.64	
Mean ± Standard Deviation ( years)	51.32 (16.61)		51.20 (16.57)		< .0001^a^
**Co-morbidity**					
Diabetes mellitus	212	15.12	253	18.05	0.0374^*^
Hypertension	565	40.3	532	37.95	0.2016^*^
Coronary artery disease	219	15.62	246	17.55	0.1704^*^
Head injury	59	4.21	83	5.92	0.0387^*^
Depression	92	6.56	119	8.49	0.0532^*^
Hyperlipidemia	295	21.04	385	27.46	< 0.0001^*^
Stroke	276	19.69	287	20.47	0.6041^*^
Mental disorders	1	0.07	1	0.07	0.99^¶^
Chronic kidney disease	26	1.85	39	2.78	0.1028^*^
Renal dialysis	1	0.07	1	0.07	0.99^¶^
**Duration between migraine date and index,** **days (mean, median)**	983 (695)		953 (663)		
**Follow time (mean, median)**	5.56 (4.91)		7.00 (6.31)		

^*^Chi-square test; ^a^*t*-test; ^¶^Fisher’s exact test.

A total of 134 subjects were newly diagnosed with dementia during the follow-up period. Table 2 displays univariate and multivariate Cox proportional hazards models for TCM users vs. non-TCM users during the years 1997–2013. After adjusting for age, sex, diabetes mellitus (DM), hypertension, coronary artery disease (CAD), head injury, depression, hyperlipidemia, stroke, mental disorder, chronic kidney disease, and renal dialysis, TCM users were significantly less likely to develop dementia (adjusted hazard ratio [aHR], 0.65; 95% confidence interval [CI], 0.46–0.95) than non-TCM users. Compared to the ≥ 80 years group, there was lower risk of developing dementia in the 40–49 years (aHR, 0.01; 95% CI, 0–0.05), 50–59 years (aHR, 0.03; 95% CI, 0.01–0.08), 60–69 years (aHR, 0.22; 95% CI, 0.13–0.38), and the 70–79 years (aHR, 0.55; 95% CI, 0.35–0.88) groups. Patients with depression had a higher risk of developing dementia (aHR, 2.43; 95% CI, 1.52–3.89) in the Cox proportional hazards model.

**Table 2:** Cox model with hazard ratios and 95% confidence intervals of dementia associated with TCM and covariates among migraine patients

Variable	No. of events (*n =* 134)	Crude*			Adjusted†		
		HR	(95% CI)	*P*-value	HR	(95% CI)	*P*-value
**TCM**							
No	66	1.00	reference		1.00	reference	
Yes	68	0.84	(0.6–1.19)	0.3277	0.65	(0.46–0.92)	0.0152
**Sex**							
Female	67	1.00	reference		1.00	reference	
Male	67	0.99	(0.71–1.39)	0.9656	0.78	(0.55–1.11)	0.1706
**Age group**							
20–29	0	-	-	-	-	-	-
30–39	0	-	-	-	-	-	-
40–49	3	0.01	(0–0.04)	< 0.0001	0.01	(0–0.05)	< 0.0001
50–59	6	0.03	(0.01–0.07)	< 0.0001	0.03	(0.01–0.08)	< 0.0001
60–69	31	0.21	(0.13–0.36)	< 0.0001	0.22	(0.13–0.38)	< 0.0001
70–79	69	0.52	(0.33–0.82)	0.0054	0.55	(0.35–0.88)	0.0134
≥ 80	25	1.00	reference		1.00	reference	
**Comorbidity (ref = no comorbidities)**							
Diabetes mellitus	48	3.16	(2.22–4.5)	< 0.0001	1.41	(0.96–2.08)	0.079
Hypertension	99	5.00	(3.4–7.36)	< 0.0001	1.07	(0.7–1.63)	0.7617
Coronary artery disease	51	3.43	(2.42–4.86)	< 0.0001	1.07	(0.73–1.57)	0.71
Head injury	7	1.15	(0.54–2.46)	0.7228	0.82	(0.38–1.77)	0.6118
Depression	23	2.85	(1.82–4.47)	< 0.0001	2.43	(1.52–3.89)	0.0002
Hyperlipidemia	51	1.97	(1.39–2.8)	0.0001	0.88	(0.6–1.3)	0.5116
Stroke	66	4.29	(3.06–6.02)	< 0.0001	1.24	(0.86–1.78)	0.2539
Mental disorders	0	-	-	-	-	-	-
Chronic kidney disease	6	2.32	(1.02–5.27)	0.0437	0.71	(0.3–1.64)	0.4191
Renal dialysis	0	-	-	-	-	-	-

Abbreviations: CI, confidence interval; HR, hazard ratio; TCM, traditional Chinese medicine.

Crude HR^*^represents the relative hazard ratio. Adjusted HR^†^ represents the adjusted hazard ratio; mutually adjusted for TCM, age, sex, diabetes mellitus, hypertension, coronary artery disease, head injury, depression, hyperlipidemia, stroke, mental disorders, chronic kidney disease, and renal dialysis in the Cox proportional hazards regression.

**Figure 2:**
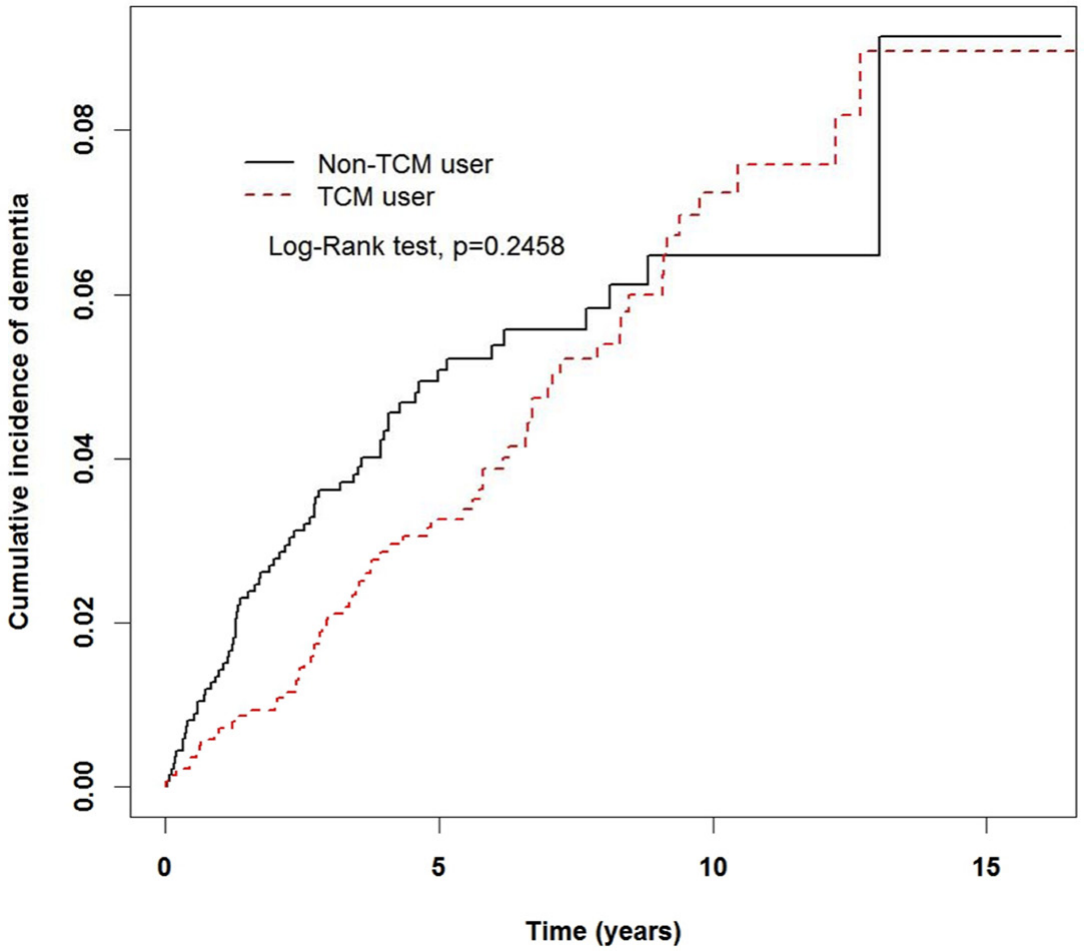
Cumulative rate of dementia in non-TCM and TCM users during the follow-up period in the migraine cohort

In Table 3, stratified by gender, the incidence rates of dementia in females and males among TCM users were 6.48 and 7.35 per 1,000 person-years, respectively; lower than the corresponding rates in the non-TCM users (9.08 and 7.83 per 1,000 person-years for females and males, respectively). In addition, female TCM users had a 0.48-fold lower risk of developing dementia than non-TCM users (95% CI: 0.29–0.81). Among the 70–79 years group, TCM users had significantly lower risk than non-TCM users (aHR, 0.6; 95% CI, 0.37–0.99). TCM users with diabetes mellitus and hypertension were less likely to have dementia than those who were non-TCM users (aHR, 0.39; 95% CI, 0.21–0.71 vs. aHR, 0.54; 95% CI, 0.36–0.82, respectively). The Kaplan-Meier analysis with log-rank test showed a lower cumulative incidence of dementia in TCM users than in non-TCM users (*P* = 0.2458; Figure 2).

**Table 3:** Incidence rates, hazard ratios, and confidence intervals of dementia for migraine patients with and without TCM use, stratified by sex, age, and comorbidity

Variables	TCM						Crude HR	Adjusted HR
	No			Yes				
	(*n =* 1402)			(*n =* 1402)				
	Event	Person- years	IR^†^	Event	Person-years	IR^†^	(95% CI)	(95% CI)
**Total**	66	7797	8.46	68	9820	6.92	0.84 (0.60–1.19)	0.65 (0.46–0.92)*
**Sex**								
Female	36	3963	9.08	31	4785	6.48	0.72 (0.45–1.17)	0.48 (0.29–0.81)**
Male	30	3834	7.83	37	5035	7.35	1 (0.62–1.63)	0.8 (0.49–1.31)
**Age group**								
20–29	0	939	0.00	0	1205	0.00	-	-
30–39	0	1450	0.00	0	1671	0.00	-	-
40–49	2	1845	1.08	1	2189	0.46	0.32 (0.03–3.58)	0.19 (0.01–3.95)
50–59	3	1558	1.93	3	1808	1.66	0.9 (0.18–4.45)	0.79 (0.15–4.17)
60–69	15	1024	14.65	16	1379	11.60	0.82 (0.4–1.66)	0.86 (0.42–1.77)
70–79	35	845	41.44	34	1311	25.93	0.64 (0.4–1.04)	0.6 (0.37–0.99)*
≥ 80	11	137	80.43	14	257	54.40	0.76 (0.34–1.68)	0.49 (0.21–1.15)
**Comorbidity**								
Diabetes mellitus								
No	40	6865	5.83	46	8143	5.65	0.99 (0.65–1.51)	0.8 (0.51–1.24)
Yes	26	932	27.90	22	1677	13.12	0.51 (0.29–0.9)*	0.39 (0.21–0.71)**
Hypertension								
No	12	5074	2.36	23	6263	3.67	1.57 (0.78–3.17)	1.02 (0.49–2.14)
Yes	54	2723	19.83	45	3557	12.65	0.67 (0.45–0.99)*	0.54 (0.36–0.82)**
Coronary artery disease								
No	42	6757	6.22	41	8228	4.98	0.82 (0.53–1.27)	0.61 (0.39–0.95)*
Yes	24	1041	23.06	27	1592	16.96	0.77 (0.44–1.33)	0.7 (0.39–1.26)
Head injury								
No	62	7521	8.24	65	9317	6.98	0.87 (0.61–1.24)	0.65 (0.46–0.94)*
Yes	4	276	14.48	3	503	5.97	0.47 (0.11–2.11)	0.3 (0.05–1.85)
Depression								
No	60	7345	8.17	51	9115	5.60	0.7 (0.48–1.02)	0.59 (0.4–0.86)**
Yes	6	452	13.27	17	705	24.12	1.92 (0.76–4.86)	1.17 (0.41–3.35)
Hyperlipidemia								
No	46	6244	7.37	37	7239	5.11	0.72 (0.46–1.11)	0.55 (0.35–0.86)**
Yes	20	1553	12.88	31	2581	12.01	0.95 (0.54–1.68)	0.78 (0.43–1.41)
Stroke								
No	34	6493	5.24	34	7892	4.31	0.84 (0.52–1.36)	0.61 (0.37–1.01)
Yes	32	1304	24.53	34	1928	17.64	0.75 (0.46–1.22)	0.66 (0.4–1.1)
Mental disorders								
No	66	7792	8.47	68	9816	6.93	0.84 (0.6–1.19)	0.64 (0.45–0.91)*
Yes	0	5	0.00	0	4	0.00	-	-
Chronic kidney disease								
No	65	7689	8.45	63	9585	6.57	0.8 (0.57–1.13)	0.62 (0.44–0.89)**
Yes	1	108	9.26	5	235	21.28	2.47 (0.29–21.23)	2.53 (0.06–115.1)
Renal dialysis								
No	66	7796	8.47	68	9812	6.93	0.84 (0.6–1.19)	0.64 (0.45–0.91)*
Yes	0	1	0.00	0	8	0.00	-	-

Abbreviations: CI, confidence interval; HR, hazard ratio; IR, incidence rates, per 1,000 person-years; TCM, traditional Chinese medicine.

Adjusted HR^†^ represents adjusted hazard ratio; mutually adjusted for TCM, age, sex, diabetes mellitus, hypertension, coronary artery disease, head injury, depression, hyperlipidemia, stroke, mental disorders, chronic kidney disease, and renal dialysis in the Cox proportional hazards regression.

*P < 0.05; **P < 0.01.

Table 4 shows that migraine patients who received only CHPs or combined CHPs with acupuncture/Tuina treatment had significantly lower risk of developing dementia than non-TCM users (aHR, 0.6; 0.39–0.92 vs. aHR, 0.62; 95% CI, 0.40–0.97, respectively). Table 5 presents the top 10 single CHPs and the top 10 formulae CHPs prescribed to migraine patients. In Table 6, the hazard ratios (HR) of the 10 single CHPs and the 10 formulae CHPs most commonly prescribed to migraine patients are shown. The majority of the single CHPs (except for Dan-Shen, Chuan-Xiong, and Jie-Geng) and all but two of the formulae CHPs (Jia-Wei-Xiao-Yao-San and Ger-Gen-Tang) were associated with significant reductions in HR.

**Table 4:** Hazard ratios and 95% confidence intervals of dementia risk associated with acupuncture/Tuina treatment among the migraine patients

	No. of events	Person- years	IR	Crude HR	Adjusted HR^¶^	Crude HR	Adjusted HR^¶^
				(95% CI)	(95% CI)	(95% CI)	(95% CI)
**Non-TCM users**	66	7797	8.46	1 (reference)	1 (reference)	-	-
**TCM users**							
Only CHPs	34	4237	8.03	0.96 (0.63–1.45)	0.60 (0.39–0.92)*	1.47 (0.90–2.40)	1.01(0.61–1.68)
Only acupuncture/Tuina	4	130	30.76	3.61 (1.31–9.90)*	2.19 (0.79–6.12)	5.62 (1.97–15.98)**	4.46(1.52–13.08)**
Combined CHPs and acupuncture/Tuina	30	5453	5.5	0.68 (0.44–1.05)	0.62 (0.40–0.97)*	1 (reference)	1 (reference)

Abbreviations: CHPs, Chinese herbal products; CI, confidence interval; HR, hazard ratio; IR, incidence rates, per 1,000 person-years; TCM, traditional Chinese medicine. ^¶^Adjusted HR represents adjusted hazard ratio; mutually adjusted for age, sex, diabetes mellitus, hypertension, coronary artery disease, head injury, depression, hyperlipidemia, stroke, mental disorders, chronic kidney disease, and renal dialysis in the Cox proportional hazards regression.

*P < 0.05; **P < 0.01.

**Table 5:** Ten most common single and formulae CHPs prescribed for migraine in Taiwan

CHPs	Frequency	Number of person-days	Average daily dose (g)	Average duration of prescription (days)
**Single CHP**				
Yan-Hu-Suo (*Corydalis yanhusuo* W.T.Wang)	1837	13416	2	7.3
Da-Huang (*Rheum palmatum* L.)	1610	12593	2	7.8
Ge-Gen [*Pueraria lobate* (Willd.) Ohwi]	1436	11376	6.6	7.9
Dan-Shen (*Salvia miltiorrhiza* Bunge)	1408	11614	3.6	8.2
Bai-Zhi [*Angelica dahurica* (Fisch. ex Hoffm.) Benth. et Hook.]	1367	9726	11.2	7.1
Zhe-Bei-Mu (*Fritillaria thunbergii* Miq.)	1278	9747	3.4	7.6
Chuan-Xiong (*Ligusticum chuanxiong* Hort.)	1254	10143	5.6	8.1
Huang-Qin (*Scutellaria baicalensis* Georgi)	1114	8291	2.3	7.4
Jie-Geng [*Platycodon grandiflorus* (Jacq.) A. DC.]	1076	7043	3.4	6.5
Gan-Cao (*Glycyrrhiza uralensis* Fisch.)	1015	7709	1.3	7.6
**Formulae CHP**				
Jia-Wei-Xiao-Yao-San	1963	16688	17.6	8.5
Chuan-Xiong-Cha-Tiao-San	1857	13049	10.4	7
Shu-Jing-Huo-Xie-Tang	1586	10547	7.5	6.7
Ji-Sheng-Shen-Qi-Wan	1290	12040	12.4	9.3
Ger-Gen-Tang	1242	8216	16.4	6.6
Ban-Xia-Xie-Xin-Tang	1235	9505	12.2	7.7
Xue-Fu-Zhu-Yu-Tang	1222	9832	7.6	8
Shao-Yao-Gan-Cao-Tang	1175	8205	5.9	7
Siang-Sha-Liu-Jun-Zi-Tang	991	8257	7.2	8.3
Zhi-Gan-Cao-Tang	942	6786	5.7	7.2

CHP, Chinese herbal product.

**Table 6:** Hazard ratios and 95% confidence intervals of dementia risk associated with CHPs used by migraine patients in Taiwan

CHPs	Dementia		Hazard ratio (95% CI)	
	*n*	No. of events	Crude*	Adjusted^†^
**Non-TCM user**	1402	66	1.00 (reference)	1.00 (reference)
**Single CHP**				
Yan-Hu-Suo	441	15	0.56 (0.32–0.98)*	0.50 (0.28–0.89)*
Da-Huang	207	5	0.40 (0.16–0.98)*	0.36 (0.14–0.93)*
Ge-Gen	328	7	0.35 (0.16–0.76)**	0.29 (0.13–0.65)**
Dan-Shen	284	9	0.54 (0.27–1.09)	0.57 (0.28–1.18)
Bai-Zhi	285	7	0.40 (0.18–0.87)*	0.44 (0.20–0.97)*
Zhe-Bei-Mu	263	6	0.37 (0.16–0.86)*	0.36 (0.15–0.86)*
Chuan-Xiong	273	13	0.76 (0.42–1.38)	0.57 (0.31–1.07)
Huang-Qin	267	2	0.12 (0.03–0.50)**	0.16 (0.04–0.65)*
Jie-Geng	248	7	0.46 (0.21–1.01)	0.46 (0.21–1.02)
Gan-Cao	255	6	0.39 (0.17–0.89)*	0.33 (0.14–0.78)*
**Formulae CHP**				
Jia-Wei-Xiao-Yao-San	362	13	0.59 (0.32–1.07)	0.69 (0.36–1.30)
Chuan-Xiong-Cha-Tiao-San	338	12	0.57 (0.31–1.06)	0.49 (0.26–0.93)*
Shu-Jing-Huo-Xie-Tang	401	15	0.60 (0.34–1.06)	0.49 (0.28–0.88)*
Ji-Sheng-Shen-Qi-Wan	179	5	0.46 (0.18–1.14)	0.35 (0.14–0.87)*
Ger-Gen-Tang	357	9	0.42 (0.21–0.84)*	0.50 (0.25–1.02)
Ban-Xia-Xie-Xin-Tang	238	4	0.27 (0.10–0.75)*	0.25 (0.09–0.71)**
Xue-Fu-Zhu-Yu-Tang	278	8	0.45 (0.22–0.95)*	0.39 (0.18–0.83)*
Shao-Yao-Gan-Cao-Tang	363	11	0.49 (0.26–0.92)*	0.42 (0.22–0.82)*
Siang-Sha-Liu-Jun-Zi-Tang	197	6	0.49 (0.21–1.13)	0.41 (0.17–0.98)*
Zhi-Gan-Cao-Tang	193	7	0.62 (0.28–1.36)	0.42 (0.19–0.94)*

Abbreviations: CHP, Chinese herbal product; HR, hazard ratio; TCM, traditional Chinese medicine.

Crude HR^*^represents the relative hazard ratio. Adjusted HR^†^ represents the adjusted hazard ratio; mutually adjusted for Chinese herb usage, age, sex, diabetes mellitus, hypertension, coronary artery disease, head injury, depression, hyperlipidemia, stroke, mental disorders, chronic kidney disease, and renal dialysis in the Cox proportional hazards regression.

*P < 0.05, **P < 0.01.

## DISCUSSION

In this nationwide cohort study, we identified migraine patients and compared the risks of developing dementia between TCM users and non-TCM users. The main findings were as follows: (1) TCM use may prevent dementia in migraine patients because it was associated with a 0.65-fold lower risk. (2) Compared with controls, older migraine patients or those with depression had a higher risk of developing dementia, but TCM use could reduce the risk of dementia in migraine patients aged 70–79 years. (3) The most common formulae CHPs prescribed were Jia-Wei-Xiao-Yao-San (JWXYS) and Chuan-Xiong-Cha-Tiao-San (CXCTS), and the most common single CHPs prescribed were Yan-Hu-Suo (*Corydalis yanhusuo*) and Da-Huang (*Rheum palmatum*).

Migraine has traditionally been considered a disorder caused by brain dysfunction and trigeminovascular nociception that does not involve structural brain abnormalities [10]. However, recent studies have shown that migraine may be a risk factor for structural changes in the brain, including increased risk of deep white matter lesions and subclinical posterior circulation infarcts [10–14]. These white matter abnormalities and silent infarct lesions may increase vascular cognitive impairment, also known vascular dementia [15]. It has also been reported that migraine patients have a higher prevalence of psychiatric comorbidities compared with the general population [16]. These psychiatric comorbidities in migraine patients, including depression, anxiety, bipolar disorder, and post-traumatic stress disorder [16], are reportedly associated with an increased risk of late-life dementia [17, 18].

As global average life expectancy increases, the number of people with dementia is expected to reach 75 million by 2030, and 131 million by 2050 [9]. Dementia will therefore have a huge economic impact in the future. Dementia is a multifactorial disorder influenced by interaction between genetic and environmental factors over the lifespan. The optimal strategy for preventing dementia is multifactorial intervention with simultaneous management of various risks, and protective lifestyle changes and pharmacological treatments [9]. Several drugs (antihypertensive drugs, statins, hormone replacement therapy, and non-steroidal anti-inflammatory drugs), and dietary and nutritional advice (Mediterranean diet, polyunsaturated fatty acids and fish-related fats, vitamins B6, B12, and folate, and antioxidant vitamins) have been proposed and investigated for dementia prevention, but the evidence to date is variable [9]. Possible reasons and limitations in these studies include different study designs, inappropriate timing and duration of interventions, and single-agent intervention in clinical trials.

The use of CAM is increasing rapidly, now exceeding a prevalence of 53% among those aged 50 years and above in the US [19]. Traditional Chinese herbal medicine is the most common CAM for the prevention and treatment of dementia in Asian countries. Some conventional drugs used for dementia originate from plants, e.g., galantamine, rivastigmine, Huperzine A, extracts of *Ginkgo biloba*, etc. [20]. Generally, TCM doctors prescribe one or two main formulae combined with several single herbs in clinical practice, as was indicated in our study. Herbal products contain complex mixtures of active components (phytochemicals), including phenylpropanoids, isoprenoids, and alkaloids [21]. Herbal medicine is reportedly associated with significant improvement in the symptoms of dementia, but it is often difficult to determine which components of the herb or herbs have biological activity [21–23]. Thus, the role of herbal medicine in the clinical management of dementia is yet to be determined. As Chinese herbal medicines contain multiple compounds and phytochemicals that may have multifaceted neuroprotective effects, they may prove beneficial in different neuropsychiatric and neurodegenerative disorders [24].

Our results are consistent with those of a previous observational study in that the most common formulae CHPs used among migraine patients in Taiwan were JWXYS and CXCTS [25]. Generally, JWXYS is used to relieve hot flushes and other menopausal symptoms, sleep disorders, and emotional disturbances [26–28]. It is of note that JWXYS is also a common formula used in patients with dementia, hypertension, and hyperlipidemia as suggested by various observational studies [29–32]. However, to date there is no relevant literature regarding the vascular effect of JWXYS on dementia or hypertension, except for one recent study that showed that it can inhibit smooth muscle cell contractility by measuring the phosphorylation of myosin light chain protein and using the collage contraction assay [30]. JWXYS reportedly inhibits the production and expression of nitric oxide, inducible nitric oxide synthase (iNOS), prostaglandin E_2_, cyclooxygenase-2, tumor necrosis factor-α, and interleukin-6 in lipopolysaccharide-stimulated RAW 264.7 macrophages [33], suggesting that it may have potent anti-inflammatory activity in the treatment and prevention of inflammatory processes or diseases. Additionally, JWXYS reportedly has antioxidant and neuroprotective effects, especially in mesencephalic dopaminergic cells, suggesting that it may be useful for the treatment of postmenopausal depression related to the degeneration of dopamine neurons [34]. In our study, nearly all the formulae were associated with significant reductions in the risk of developing dementia. Despite a lack of studies on their neuroprotective effects, several herbs among these formulae have been shown to have sedative, antioxidant, or anti-inflammatory effects, including *Glycyrrhiza uralensis* (Gan-Cao), *Angelicae sinensis* (Dang-Gui), and *Ligusticum chuanxiong* (Chuan-Xiong) [25]. Further studies may focus on the antioxidant or anti-inflammatory activities exerted by specific molecules present in prescribed herbal medicines.

In this study, most of the commonly used single CHPs were associated with a significant reduction in the risk of developing dementia in migraine patients, except Dan-Shen, Chuan-Xiong, and Jie-Geng. The most frequently prescribed single CHP, Yan-Hu-Suo (*Corydalis yanhusuo*), is used in TCM for pain relief and blood activation. L-tetrahydropalmatine (L-THP), identified as one of the major active components of Yan-Hu-Suo, has been used to treat headache and chemotherapy-induced pain, and also exerted a significant antinociceptive effect on chronic inflammatory and neuropathic pain in a mouse model, without associated motor deficits [35]. Dehydrocorybulbine, another active component of Yan-Hu-Suo, is also reportedly effective against inflammatory pain and injury-induced neuropathic pain [36]. Acetylcholinesterase (AChE) inhibitors are widely used for the symptomatic treatment of Alzheimer’s disease (AD) and other dementias. Previous research has shown that compounds isolated from Yan-Hu-Suo, including berberine, palmatine, jatrorrhizine, coptisine, and dehydrocorydaline, had dose-dependent inhibitory effects on AChE activity [37]. Increasing evidence demonstrates that beta-amyloid (A-beta) elicits oxidative stress, which contributes to the pathogenesis and progression of AD [38]. Thus, there is interest in developing antioxidant therapies for the prevention or treatment of cognitive decline during AD. Rhein, puerarin, and imperatorin, which are major medicinal ingredients isolated from Da-Huang (*Rheum palmatum*), Ge-Gen (*Pueraria lobata*), and Bai-Zhi (*Angelica dahurica* ), respectively, reportedly exert antioxidant effects [39–41]. Another single CHP, Dan-Shen (*Salvia miltiorrhiza*), a well-known TCM herb used for the treatment of cerebrovascular diseases including stroke, has also been shown to have positive effects in neurodegenerative diseases. The main bioactive constituents of Dan-Shen are the lipophilic diterpenic quinones known as tanshinones, and the hydrophilic depsides known as salvianolic acids [42]. Both tanshinones and depsides can protect against A-beta-induced toxicity, and have anti-inflammatory activity [42]. Tanshinone IIA has been shown to reduce the risk of AD by inhibiting iNOS, matrix metalloproteinase-2, and nuclear transcription factor kappa transcription and translation in the temporal lobes of rat models of AD [43].

The occurrence of AD and other dementias is higher in women than in men, particularly in the most elderly, and the burden of dementias is greater for women than for men [44]. In our study, gender was not a significant risk factor for developing dementia among migraine patients. The female TCM users had a 52% lower risk compared with non-TCM users. TCM may be more effective in females than in males. The cause is unknown, and possible reasons include genetic factors, hormonal factors, and/or a higher prevalence of unhealthy lifestyles in men such as smoking and alcohol use. The risk of developing dementia increased with age in migraine patients, but only patients aged 70 to 79 years who used TCM had a significant reduction in HR. A possible reason is that early onset dementia may be more attributable to traumatic brain injury, alcohol use, human immunodeficiency virus, and frontotemporal lobar degeneration than late onset dementia [45]. TCM may be more effective in the prevention of late onset dementia that is mainly caused by AD, but not in those older than 80 years.

In our clinical practice, migraine patients received TCM treatment including Chinese herbal medicine, acupuncture, and Tuina. Acupuncture has been reported to improve cognitive function in those with dementia by regulating glucose metabolism, enhancing neurotransmission and reducing oxidative stress, Aβ protein deposition, and neuronal apoptosis in animal studies [46, 47]. In our study, migraine patients who accepted CHP treatment alone, or that combined with acupuncture/Tuina, had significant reduction in HR compare to non-TCM users. TCM users who accepted only acupuncture/Tuina treatment had no significant reduction in HR compared to non-TCM users. Therefore, acupuncture/Tuina did not have a preventive effect on the development of dementia in our study. However, the sample of patients who received only acupuncture/Tuina was small and sampling bias may have occurred. Larger clinical trials should be designed to determine the effects of acupuncture/Tuina on dementia development.

The strengths of this study include the immediate availability and comprehensiveness of the nationwide database. In addition, this 15-year follow-up study allowed us to examine the use of TCM confidently with regard to associations between migraine and the risk of dementia over a long latency period. Despite these strengths, several limitations should be noted when interpreting the results of the present study. First, the identification of TCM exposure and outcomes were based on ICD-9-CM codes, and misclassification is a possibility. To minimize this potential error, we selected subjects with either migraine or dementia only after they were recorded as having at least two ambulatory or inpatient claims reporting consistent diagnoses. Second, information on lifestyle factors, education levels, and nutritional factors were not available from the National Health Insurance Research Database (NHIRD). Sedentary lifestyle, smoking, heavy alcohol consumption, lower education level, and deficiencies in vitamins B6, B12, D, and folate are associated with an increased risk of dementia [9]. The failure to adjust for putative risk factors may have resulted in biased estimates of risks of dementia in our sample. Third, we were not sure whether the patients exactly took medication as their physicians prescribed or not. Other preparations of Chinese herbal remedies, health foods containing natural herbs, folk medicine, and direct purchases from TCM herbal pharmacies are not reimbursed by NHI, and therefore, were not analyzed in this study. However, the high healthcare insurance coverage and low prices of government-approved CHPs have led to a reduction in herbal folk medicine use. Furthermore, prescriptions for medications issued before 1996 were not reflected in the data analysis in the present study. This omission could possibly result in underestimating cumulative frequencies, and may have weakened the effect of the specified CHPs.

In conclusion, this 15-year follow-up cohort study found that the use of TCM during the treatment of migraine was associated with a 35% lower risk of developing dementia compared with the risk among non-TCM users. This finding was statistically significant, and could serve as a strong reference for healthcare providers to help establish more effective therapeutic interventions to improve the prognosis of patients with migraine.

## MATERIALS AND METHODS

### Data sources

Taiwan’s compulsory universal NHI program was developed in 1995 by the NHI Administration (NHIA), and provided coverage to more than 23.03 million residents in Taiwan at the time. In 2008, > 99% of the Taiwanese population was enrolled in the NHI program. Reimbursed TCM services included CHPs, and acupuncture or moxibustion in ambulatory clinics. The database (http://nhird.nhri.org.tw/date_01_en.html) contains all longitudinal reimbursement information on sex, birth date, medications, and diagnosis codes based on the International Classification of Disease, Ninth Revision, Clinical Modification (ICD-9-CM). We used the Longitudinal Health Insurance Database 2000 (LHID 2000), which contains medical information on 1 million beneficiaries randomly sampled from the registry of all beneficiaries in 2000. As a group, the sampled patients exhibit no significant differences in age, sex, birth year, or average insured payroll-related amount from the general population. The requirement for informed consent was waived because the National Health Insurance Research Database contains anonymized secondary data for research. This study was approved by the Institutional Review Board of China Medical University (CMUH104-REC2-115).

### Study population

Patients who were newly diagnosed with migraine between 1997 and 2010 were identified as the migraine cohort. The population with migraine (*n* = 36,865) were required to have had at least two ambulatory or inpatient claims with a diagnosis of ICD-9-CM code 346, from 1997 to 2010. We excluded patients younger than 20 years, those who had withdrawn from the NHI program within a year of follow-up, and those diagnosed with dementia before their initial diagnosis of migraine. We also excluded those who utilized TCM treatment before the initial diagnosis of migraine, but did not utilize TCM treatment during the follow-up period. For each category of migraine patients, those who had at least one TCM outpatient clinical record were defined as TCM users during the follow-up period (*n* = 28,456), whereas those who had no TCM outpatient records were defined as non-TCM-users (*n* = 1,656; Figure 1). In the TCM patient group, the index date was the first TCM treatment utilized after the initial diagnosis of migraine. In the non-TCM group, no index date for the first TCM treatment could be assigned. Thus, we randomly assigned a “pseudo diagnostic date” to each patient within the initial diagnosis date of migraine and the endpoint, as the index date for that group. The same eligibility criteria were applied to each group, yet the distributions of age and sex were unbalanced between groups. To balance comparability between the TCM and non-TCM groups, we randomly selected equal numbers from each group and compared the subgroups compiled based on combinations of age (5-year increments), sex, index year, and year of migraine diagnosis.

### Outcome

The primary outcome was the occurrence of dementia, defined as the first ambulatory event or hospitalization with an ICD-9-CM code of 290.0–290.4, 294.1, 331.0, or 331.1–331.2, diagnosed by a neurologist, neurosurgeon, or psychiatrist during the follow-up period. Both cohorts were followed until December 31, 2013.

### Covariate assessment

Sociodemographic factors included age and sex. Age was initially divided into 7 groups: 20–29 years, 30–39 years, 40–49 years, 50–59 years, 60–69 years, 70–79 years, and ≥ 80 years. Baseline comorbidities were considered to be present if ICD-9-CM codes appeared two or more times in the outpatient or inpatient claims before the initial diagnosis of migraine, and included diabetes mellitus (ICD-9-CM code 250), hypertension (ICD-9-CM code 401), coronary artery disease (CAD; ICD-9-CM codes 410–414), head injury (ICD-9-CM codes 850–854 and 959.01), depression (ICD-9-CM codes 296.2, 296.3, 300.4, and 311), hyperlipidemia (ICD-9-CM code 272), stroke (ICD-9-CM codes 430–438), mental disorder (ICD-9-CM codes V11, V79.9, and 310.9), chronic kidney disease (ICD-9-CM codes 585–586 and 403–404), and renal dialysis (ICD-9-CM codes V45.1 and V56).

### Statistical analysis

Differences in demographic characteristics and comorbidities between the TCM and non-TCM groups were examined using the chi-squared test and two-sample *t*-test. Univariate and multivariate Cox proportional hazards models were used to evaluate the hazard ratios for dementia in the TCM group. The difference in the development of stroke between the two groups was estimated using the Kaplan-Meier method and the log-rank test. Statistical analysis was performed and figures were created using SAS 9.4 (SAS Institute, Cary, NC, USA) and R software. Statistical significance was defined as *P* < 0.05 in two-tailed tests.

## Abbreviations

Ach: Acetylcholinesterase
AD: Alzheimer’s disease
CAD: coronary artery disease
CAM: complementary and alternative medicine
CHP: Chinese herbal product
CI: confidence interval
CXCTS: Chuan-Xiong-Cha-Tiao-San
DM: diabetes mellitus
HR: hazard ratio
ICD-9-CM: International Classification of Diseases 9th Revision Clinical Modification
iNOS: nitric oxide synthase
JWXYS: Jia-Wei-Xiao-Yao-San
LHID: Longitudinal Health Insurance Database
L-THP: L-tetrahydropalmatine
NHI: National Health Insurance
NHIRD: National Health Insurance Research Database
NTD: New Taiwan Dollars
OR: odds ratio
TCM: traditional Chinese medicine
